# Automatically Detected Pecking Activity in Group-Housed Turkeys

**DOI:** 10.3390/ani10112034

**Published:** 2020-11-04

**Authors:** Jennifer J. Gonzalez, Abozar Nasirahmadi, Ute Knierim

**Affiliations:** 1 Farm Animal Behaviour and Husbandry Section, University of Kassel, 37213 Witzenhausen, Germany; uknierim@uni-kassel.de; 2 Agricultural and Biosystems Engineering Section, University of Kassel, 37213 Witzenhausen, Germany; abozar.nasirahmadi@uni-kassel.de

**Keywords:** automatic detection, cannibalism, environmental enrichment, pecking activity, poultry, welfare

## Abstract

**Simple Summary:**

Cannibalism is one of the biggest welfare issues of today’s turkey husbandry. We hypothesized that changes in pecking activity might indicate imminent cannibalism. Therefore, in this pilot study a newly developed automatic pecking activity detection was validated, and continuously applied to gain information about pecking activity of group-housed turkeys during the rearing phase and before a cannibalistic outbreak. The pecking object was used by turkeys the whole recording time. Activity on the object was highest in the morning. No clear trend in pecking activity development before an outbreak has yet been found. Pecking detection has to be further tested under various farm conditions. The system can be used in further research in order to survey changes in pecking activity in turkeys.

**Abstract:**

In search for an early warning system for cannibalism, in this study a newly developed automatic pecking activity detection system was validated and used to investigate how pecking activity changes over the rearing phase and before cannibalistic outbreaks. Data were recorded on two farms, one with female (intact beaks) and the other with male (trimmed beaks) turkeys. A metallic pecking object that was equipped with a microphone was installed in the barn and video monitored. Pecking activity was continuously recorded and fed into a CNN (Convolutional neural network) model that automatically detected pecks. The CNN was validated on both farms, and very satisfactory detection performances were reached (mean sensitivity/recall, specificity, accuracy, precision, and F1-score around 90% or higher). The extent of pecking at the object differed between farms, but the objects were used during the whole recording time, with highest activities in the morning hours. Daily pecking frequencies showed a low downward trend over the rearing period, although on both farms they increased again in week 5 of life. No clear associations between pecking frequencies and in total three cannibalistic outbreaks on farm 1 in one batch could be found. The detection system is usable for further research, but it should be further automated. It should also be further tested under various farm conditions.

## 1. Introduction

Poultry meat production increased between 1961 and 2017 from nine to 122 million tons due to increasing demand [1]. In 2018, the share of turkey meat was 4.6%, with 5.9 million tons produced worldwide [2]. At the same time, consumers demand higher farm animal welfare standards, especially in middle and northern Europe e.g., Germany [3]. Turkey husbandry is saddled with many welfare challenges, for example, cannibalism and severe feather pecking, which are abnormal redirected behaviors [4,5]. Cannibalism is one of the major issues and it is defined as the pecking of the skin of a conspecific, which leads to wounds and the intake of blood or tissue by the pecking animal [6]. Cannibalism can spread rapidly throughout the herd [6] and it often results in the death of the affected animals [6,7]. It is also a major economic problem for poultry farmers [8] and it can occur at any time, both during rearing and fattening. Birds with pecking injuries were found as early as the first weeks of life [7,9,10]. As a preventive intervention beak trimming is generally used. However, it does not remedy the cause, but only minimizes resulting injuries. Beak trimming is a highly criticized practice regarding animal welfare. It is a painful practice that has an influence on beak related behavior [11,12]. Hence, it is discussed to be banned in Germany [13,14]. Should the ban be approved, farmers will likely encounter higher numbers of injured and/or dead animals [5,15]. One possibility for minimizing the problem could be the detection of imminent cannibalism to prevent it from spreading. For this, a reliable indicator and practical way to assess this indicator needs to be found. Looking at the case of tail biting in pigs, indications of restlessness before tail biting were found by Statham et al. [16] (more standing, less sitting or lying inactive four days before a tail biting event) and Zonderland et al. [17] (more posture changes, increase in total activity). Additionally, turkey farmers often report an increase in bird activity before a cannibalistic outbreak. A considerable proportion of activity accounts for beak related behavior (45% in week 2 to 28% in week 11 of life), which consists of feeding, drinking, preening, and environmental and bird pecking [18]. Moreover, nutrient deficiencies were found to increase the exploratory behavior on an individual level, which can be redirected towards other birds [19] (for domestic fowl). Studies on an individual, genetic level yielded heterogeneous results on the possible relationships between foraging or explorative pecking and redirected pecking. Cloutier et al. [20] (for laying hens) and Busayi et al. [21] detected no association between pecking at inanimate objects (object pecking) in a test situation and feather pecking or cannibalism. In contrast, Rodenburg and Koene [22] found laying hens selected for low feather pecking to perform more ground pecking than lines selected for high feather pecking. However, no study looked yet at longitudinal turkeys’ pecking activity changes on the group level in relation to occurrences of cannibalism.

Studies on pecking behavior in turkeys have mainly been done by direct observation and under experimental conditions [18,23,24,25]. Busayi et al. [21] used a Peckometer to assess the pecking activity (pecks and pulls) of two different turkey breeds in an experimental pen. The Peckometer consisted of feathers in a resilient holder. Oscillations that resulted from pulling or pecking were recorded by strain gauges. This approach was not further investigated. Further, the Peckometer was never used to continuously record pecking under near farm conditions. However, in this way, subtle changes for example of pecking activity would be impossible to detect. Automatic systems gained popularity and importance over the last years. They are increasingly used for recording welfare measures in farm animal husbandry. One recording method is audio analysis. This method is for example used in poultry for the monitoring of feed intake [26], disease [27,28], thermal comfort [29], and abnormal vocalization [30,31]. Bright [31], for example, found laying hen flocks with feather pecking to perform significantly more startle/pain squawks per second and showed overall more vocalization per second than non-feather pecking flocks. In this study, spectrograms and visual inspection were used in order to assign call types. Aydin et al. [26] linked the pecking sound of 12 individual broilers with the total feed intake. They were able to identify pecking sounds with an accuracy of 93% and found a strong correlation between pecking and feed intake. However, audio analysis also has the potential to detect pecking activity in a flock at an inanimate object.

The objective of this work was to test a newly developed automatic detection system for pecking activity of turkeys based on audio data analysis [32] under near-commercial conditions. The aims were to (1) validate the system, (2) explore pecking activity at an inanimate non-edible object in the long-term during the rearing phase, and (3) test for interrelations between pecking activity and cannibalistic events.

## 2. Materials and Methods

### 2.1. Equipment for Data Recording

Two newly developed pecking objects (PO, stainless steel balls, diameter of 130 mm) were suspended from the ceiling and positioned around 20 cm above the ground. Each ball had a built-in microphone (Monacor VB-120MIC, bit rate of 128 kbps, and audio sample rate of 44,100 Hz) and, for validation purposes, was connected via cable to a camera (TosiNet Realtime 2K 4MP PoE-IP-camera, frame rate of 20 fps). The camera was positioned on the ceiling with its lens pointing downward and directly above the ball. The height of the POs was adjusted every week based on the growth and height of the birds. They were supposed to reach the middle of the ball easily with their beak.

### 2.2. Data Processing

Recorded audio data were extracted from video footages and sampled in 1 s slots at 44.1 kHz. Surrounding noise (e.g., ventilation and heating systems, bird vocalization, and other low-frequency ambient sounds) were eliminated by a high-pass filter (stopband attenuation = 36 dB, Steepness = 0.8, minimum-order infinite impulse response filter) with a cut-off frequency of 1.6 kHz [32]. The filtered sound files were fed into a convolutional neural network (CNN) model. The detection method was developed in MATLAB 2018b (MathWorks^®^). For more details on the development of the used CNN-model, see Nasirahmadi et al. [32]. The model detected pecks on a one second basis, regardless of how many pecks happened in that second, and they were coded as 1 for “peck” and 0 for “non-peck” in Excel files (.xlsx). Altogether, around 2,376,000 million data points consisting of either 1 or 0 were analyzed for this study.

### 2.3. Farm 1

#### 2.3.1. Animal and Housing

The experiment was conducted on a German research farm (Farm for Education and Research in Ruthe of the University of Veterinary Medicine Hannover, Foundation) during two rearing and fattening periods with a group of 2170 female turkeys (B.U.T. Big 6) with intact beaks. From day one, the birds were housed in a Louisiana type barn (29.2 × 15.9 m, 320 m^2^) that was equipped with natural and forced ventilation systems. The birds were fed according to a multiphase feeding program and provided with wood shavings as bedding material. A light program with artificial light sources (at least 20 lux) in addition to natural light supplied light for not more than 16 h. In the case of cannibalism, the light was dimmed to under 20 lux for a short period of time. Lighting, feeding, temperature and other husbandry regimes were in accordance with German practice recommendations [33]. The birds stayed in the same barn during rearing and fattening. For the first seven days, turkey chicks were reared in smaller groups in chick guards (plastic rings) in order to keep them close to the heater, feed and water and minimize animal losses. After the chick guards had been opened, birds had unrestricted continuous access to the two pecking objects (PO1 and PO2) at different places in the barn. The birds were provided with different kinds of environmental enrichments at all times and additionally when required, for example, after a cannibalistic outbreak. Enrichment consisted of e.g., egg cartons, hanging metal pieces, paper towels, or popcorn.

#### 2.3.2. Validation

One rearing and fattening period was used in order to validate detection performance. Data were available from week 2 until 14 of life (lacking data for week 15–16 due to technical problems). Randomly selected 300 s per week were fed into the CNN-model for detection. During the same time, one-zero behavior sampling [34] per second was carried out from video, i.e., for each second it was noted whether pecks occurred or not. The trained observer reached a very good intra-observer reliability (PABAK = 0.94). The evaluation parameters were calculated.

#### 2.3.3. Data Recording

A rearing and fattening period under similar conditions was used to continuously record pecking activity. Videos and sounds were continuously recorded for 14 weeks (week 2–15 of life; missing data for week 16 due to technical difficulties). Data were analyzed from 7 a.m. until 7 p.m. (12 h) during the full rearing period (week 2 to 5, day of live 8–35). This covered a great part of the light period (6 a.m.–10 p.m.). Further, data for three cannibalistic events were analyzed. Nine days before a cannibalistic outbreak and the first day of the outbreak were included in the analysis.

Farm documentation included number of birds separated or killed per day and probable cause, particularly in the case of pecking injuries, daily times of animal controls or entering the pen for other reasons (e.g., provision of litter or enrichments). When, at minimum, nine birds were killed or separated due to pecking injuries within 24 h, it was regarded a cannibalistic outbreak and farm practice reported the incident (modified after Kulke et al. [10], with a slightly lower number than the proposed 0.5% of injured animals, to ensure early detection). This criterion was fulfilled three times, in week 4, 10, and 15 of life.

### 2.4. Farm 2

#### 2.4.1. Animal and Housing

The second part of the experiment was conducted on a commercially managed farm in a group of 6450 male turkeys (B.U.T. Big 6) with trimmed beaks. From day one, the turkeys were housed in a closed barn with forced ventilation. Rearing took place in a barn where females were also reared in a separate section. Thereafter the males were transferred to a similarly equipped fattening barn. The birds were fed according to a multiphase feeding program and provided with straw as bedding material. A light program with artificial light sources (at least 20 lux) in addition to natural light supplied light for not more than 16 h. In the case of cannibalism, the light was dimmed to under 20 lux. Stocking density, lighting, feeding, temperature, and other housing systems were in accordance with German practice recommendations [33]. From day one, the chicks had unrestricted, continuous access to the entire barn and the pecking object. This pen was equipped with one pecking object in the middle of the pen. The birds were not provided with changing environmental enrichment.

#### 2.4.2. Validation

Two weeks during the rearing period were validated in order to verify that the detection system could reliably detect pecking activity under different environmental and management conditions without re-training and using the same CNN model. For week 4 and 5 of live 120 s were randomly selected and fed into the model for detection. Validation was conducted, as described above.

#### 2.4.3. Data Recording

Video and audio data were continuously recorded during the rearing period (week 2 to 5, day of life 8–35). Data were analyzed from 7 a.m. until 7 p.m. (12 h). No cannibalistic outbreak occurred during the rearing period according to farm documentation.

### 2.5. Statistical Analysis

For descriptive statistics and statistical analysis, the R-Software was used (R Core Team, 2020). Sensitivity/recall, specificity, precision, accuracy, and F1-score (Table 1) were calculated for validation data. Due to non-normal distribution of the data (Shapiro-Wilk Test), Spearman correlation analyses were performed concerning the interrelation between pecking activity at PO1 and PO2 on farm 1, between mean daily pecking activity and day of life on both farms, as well as between the mean daily pecking activity and number of injured animals on farm 1. To test for differences in pecking activity during different times of day, data of the whole rearing period for each farm were categorized into morning (7–11 a.m.), noon (11 a.m.–3 p.m.), and afternoon (3–7 p.m.), and then compared using the Friedman rank sum test.

## 3. Results

### 3.1. Validation of Detection System

For most parameters values of 80% and higher were reached, with only single lower values: on farm 1 in four weeks for precision (lowest: 44.4% in week 8) and for F1-scores (lowest: 50.0% in week 8) and in two weeks for sensitivity (lowest: 57.1% in week 8), and on farm 2 for sensitivity (74.2%) in week 4 (Table 2). Summarized over all weeks, the detection performance on farm 1 was around 90% and higher for all parameters (Table 2).

### 3.2. Pecking Activity over the Rearing Period

**Figure 1:**
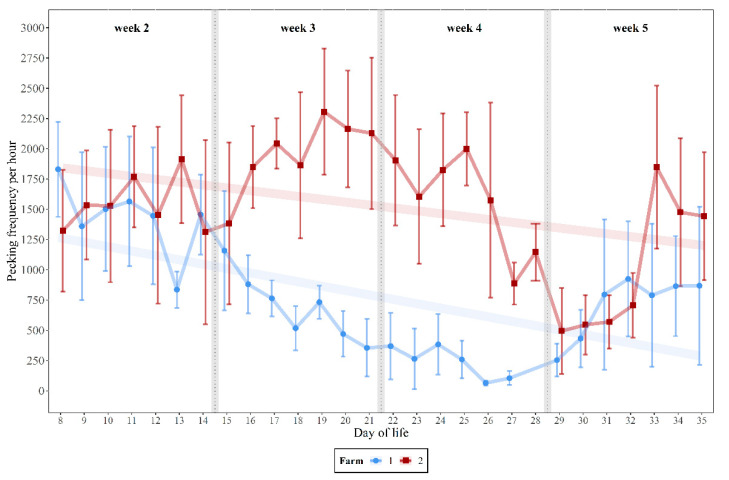
Mean values and standard deviations of pecking frequencies per hour for life day 8–35 on farm 1 and 2 (*n* = 12 h) and Spearman correlations (watermarks) with day of life (*n* = 28 days; farm 1: missing value on day 28).

The pecking frequencies showed similar trends at both pecking objects (Spearman correlation analysis: r = 0.751, *p* < 0.001, *n* = 27, one missing value). Mean pecking frequencies varied on both farms over the 28 days (LW 2–5) of the rearing period. On farm 1, the mean pecking frequencies per hour at both pecking objects over the 12 h recording time had the largest fluctuations in weeks 2 and 5. In weeks 3 and 4, the frequency of pecking decreased steadily, down to a mean of 64.3 (median 56) pecks/h on day 26, when a cannibalistic outbreak was reported. The standard deviation also decreased during this time (Figure 1). On farm 2, a higher mean pecking activity, fluctuation, and standard deviation could generally be observed. From the fourth week of life onwards pecking activity decreased and reached its lowest value on the 29th day with an average value of 494.16 (median 380) pecks/h. On both farms, the mean pecking activity showed moderate and low negative correlation with increasing age (Spearman correlation: R_farm1_ = −0.519, *p* < 0.001; R_farm2_ = −0.256, *p* < 0.001), although the pecking activity increased again in week 5 (Figure 1).

### 3.3. Variation in Pecking Activity over the Day

**Figure 2:**
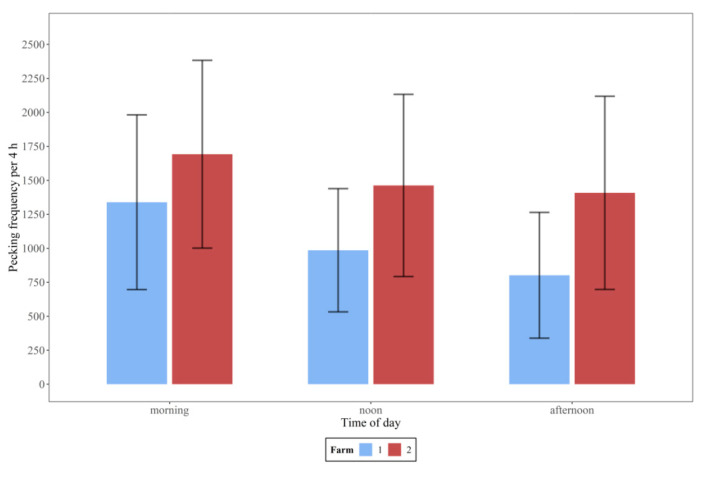
Mean pecking frequencies (and standard deviation) in the morning, at noon and in the afternoon (each over 4 h, *n* = 28 days) on farm 1 and 2.

Figure 2 shows the differences between mean pecking frequencies during morning, noon, and afternoon on both farms. With regard to the daily distribution, the highest pecking activity on both farms was in the morning, decreasing during the day until afternoon (Friedman rank sum test, *p* < 0.001, *n* = 28 days).

### 3.4. Pecking Activity and Cannibalistic Events

**Figure 3:**
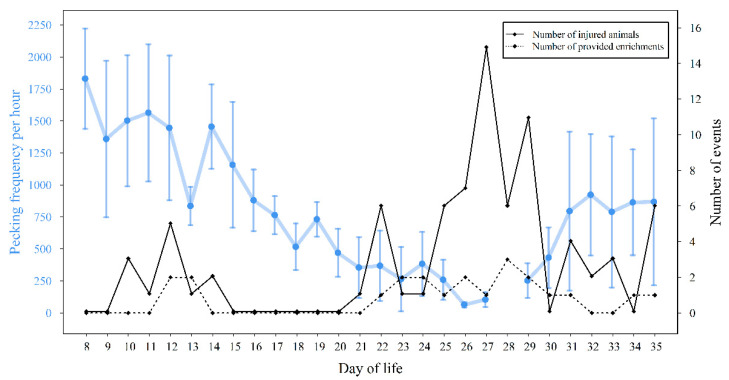
Mean values and standard deviations of pecking frequencies per hour (blue) for life day 8–35 on farm 1 (*n* = 12 h, day 28: missing value), number of injured animals (black) separated and the number of various enrichments offered from day 8 to 35 of life.

First birds with pecking injuries were recorded on the 10th day of life. The first cannibalistic outbreak was reported on day 26 (six birds with injuries on day 25 and seven birds with injuries the next morning). Over the whole rearing period, there was a trend for a low negative correlation between pecking frequency and numbers of injured animals (Spearman correlation: R =−0.352, *p* = 0.072, *n* = 27 days). At the same time, the number of different enrichments provided increased following an increase in the number of injured animals (Figure 3).

**Figure 4:**
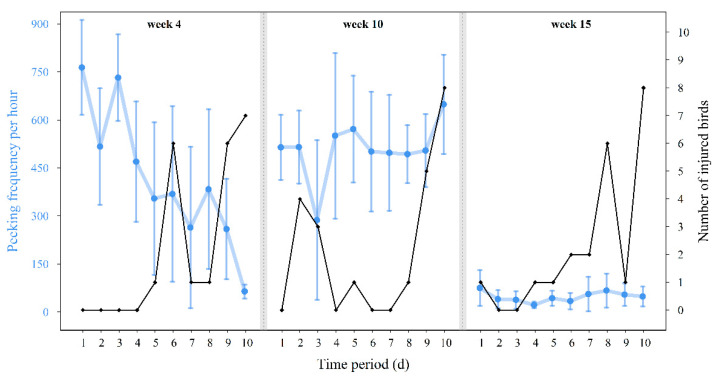
The development of mean values and standard deviations of pecking frequencies per hour (blue) from 9 days prior cannibalistic outbreaks and the outbreak day in weeks 4, 10, and 15 of life and numbers of injuries for each day (black, *n* = 12 h).

Figure 4 shows the developments of pecking frequency from nine days before until the outbreak day for all cannibalistic outbreaks. They showed all different frequency developments before a cannibalistic outbreak. While, before the earliest outbreak in week 4, a clear downward trend is noticeable (Spearman correlation: R_week4_ = −0.903, *p* < 0.001, *n* = 10 days), before the other two outbreaks no significant correlations between pecking activity and day of life were present (R_week10_ = 0.042, *p* = 0.919; R_week15_ = 0.20, *p* = 0.583, *n* = 10 days). Similarly, the numbers of injured animals separated and pecking activity were highly negatively correlated in week 4 (Spearman correlation: R_week4_ = −0.889, *p* < 0.001, *n* = 10 days), but not in weeks 10 and 15 (R_week10_ = 0.232, *p* = 0.519; R_week15_ = 0.346, *p* = 0.327, *n* = 10 days).

## 4. Discussion

### 4.1. Validation of Detection System

For this validation study, the data were recorded on two farms with different husbandry systems. The differences in sex, beak treatment, housing, and group size allowed for testing the functionality of the detection system under various conditions in order to ensure usability on different farms. In general, the pecking detection with the trained CNN model showed good results, with an overall performance of around 90% and higher, although differences between the weeks of life occurred (Table 2). The challenges for the assessment of the audio data were the great amount of noise sources in and surrounding the farm environment. Sources of noise included vehicle transportation, ventilation, heating, feeding system, stockperson activities, and birds’ vocalization. Those noises may overlap with the sound caused by the peck that initially created detection problems that we solved by filtering [32]. Further, pecking intensity changed with the age and growth of the birds. The constant change of surrounding noises and the changes of pecking intensity over the rearing and fattening period may lead to misclassifications of the model [35]. With increasing age of the birds, the number of incorrectly classified non-pecks as pecks slightly increased. This was mainly due to strong pecking towards further metal objects, i.e., pipes and feeders by the turkeys and the model wrongly detected those events as pecks at the object. In addition, when only low numbers of pecks were observed in the videos e.g., in week 8 with only seven pecks per 300 s, misclassifications had a big impact. Thus, three misclassifications led to a low sensitivity in week 8, with only 57.1%. The same issue occurred in week 11 and 12 of life. Despite the challenges, and with very few exceptions, the system performed well. The second validation on farm 2 (Table 2) confirmed the good detection performance, even though the environmental conditions, sex, and state of the beak were different and the model was not newly trained for those new conditions. This validation could be an indicator that the system could be used on other farms without long training and validation of the system, but this has to be tested further under different farm and management conditions.

### 4.2. Pecking Activity over the Rearing Period

Both of the pecking objects showed similar trends in recorded pecking frequencies, which were therefore summed. However, the detection objects (microphone and metallic ball) are rather affordable; it creates no large financial burden to install more than one PO in the barn. The overall pecking activity decreased in general over the rearing period on both farms. This conforms to other studies that report a decreasing usage of enrichments in the first week after availability [36]. It is expected that with decreasing novelty their attraction decreases. However, most observations of the use of enrichment objects are relatively short-term. In our long-term recording we could detect increased pecking activity again in week 5 of life, and still high frequencies in week 10 of life. It is possible that enrichment objects regain attractiveness from time to time. However, in this study, the pecking object was not supposed to act as an effective environmental enrichment, but instead as part of the normal barn environment on which pecking activity might be differentially directed, depending on the current pecking motivation of the birds. Nevertheless, the stainless steel ball was chosen (besides suitability for disinfection and durability) in order to secure sufficient attention of the birds. Evidently, shiny objects, like nails and rivets, are attractive to turkeys [24]. Cannibalism could have an influence on the pecking activity and thus on the negative correlation between pecking activity and day of life on farm 1. For that reason, the interrelation was also tested without the days before and on the cannibalistic outbreak. However, because of the remaining negative correlation, those days were not taken out from our analysis. In general, the pecking activity was higher on farm 2. Besides a possible difference in pecking activity between male and female turkeys and potential group size effects, the offer of enrichment could have had an influence. The female birds on farm 1 were provided with various changing enrichments on which pecking could be directed. On farm 2 no changing enrichment was provided which could lead to a higher concentration of pecking towards one pecking object. The pecking activity on farm 2 also showed stronger standard deviations and fluctuations. This farm did not report any outbreaks of cannibalism during the rearing period. A statement regarding possible correlations between the conspicuous drop in activity at the pecking object at the end of week 4 and until day 29 and other behavior cannot be made because no corresponding data are available. However, this study provides first detailed and long-term data on pecking behavior of turkeys at an inanimate, non-edible object in early life, which can be an important step for learning more about beak related (normal and abnormal) behavior of turkeys.

### 4.3. Variation in Pecking Activity over the Day

On both farms, we found the highest pecking activity in the morning with the start of the light period and a decrease during the day. Bircher and Schlup [23] observed seven to 14 weeks old turkeys (B.U.T. Big 6) in an extensive and enriched environment (e.g., perches and outdoor run) and found foraging behavior that included object and substrate pecking to be the highest in the first four hours of observation which similarly were from 7–11 a.m. During the rest of the day, proportions of time spent foraging stayed the same. However, recording by Bircher and Schlup [23] based on visual observation could have missed small changes in pecking activity during the day. Additionally, they included foraging behavior, not just object pecking, which overall could stay the same over the day, and genetic and environmental differences may also contribute to differing results. Hughes and Grigor [18] reported higher environmental pecking when feeding activity was high, which suggests some interrelation between foraging and environmental pecking and could explain higher pecking activity on the pecking object during the morning. This must be further investigated in more flocks and under different farm environments. The detection system could, for instance, also be used to assess changes in pecking activity between different feeding regimes.

### 4.4. Pecking Activity and Cannibalistic Events

On day 26 of life (day 19 of recording), the first cannibalistic outbreak was reported from farm 1. It is not unusual that the first outbreak occurs as early as in the rearing phase [7,9,10] which renders that period especially important regarding management and animal control in order to prevent or minimize cannibalism. Before the outbreak, pecking activity on the objects steadily declined and it was the lowest on the actual day of outbreak. Furthermore, pecking activity and injured birds separated by farm personnel were negatively correlated during the ten days before this cannibalistic outbreak. Such associations might be explained by the increased redirection of environmental pecking towards pen mates, in line with indications in domestic fowl that birds who feather peck are more active and spend less time with food pecking and environmental pecking [37]. It would also be consistent with the notion that feather pecking likely is misdirected ground pecking [38]. The results regarding domestic fowl are not completely applicable to turkeys, but redirected foraging behavior is also considered to be the underlying motivation for severe feather pecking in turkeys [39]. However, later outbreaks did not show similar associations. Moreover, before the first outbreak, there was a possible confounding due to the provision of new enrichments following the separation of higher numbers of injured birds as a management measure. Pecking activity at the object may have been reduced because it was redirected to the new enrichments. Conversely, the pecking activity at the object may reflect the attractiveness of the current enrichment, which should be investigated further, also in the light of the pronounced differences in pecking frequencies between the two farms investigated, although they may have been influenced by further factors such as sex of the birds.

At the same time, it must be questioned whether the applied definition of outbreaks of cannibalism was appropriate, particularly in the context of early detection. Cannibalism can occur at an early stage (the first animals separated or killed due to injury as early as on the 10th day of life) and afterwards more or less birds were separated because of pecking injuries every day. Therefore, it is possible that, regardless of the fluctuations in numbers of injured birds, cannibalism stays in the flock once it first occurred without disappearing completely.

The newly developed long-term monitoring tool now opens new possibilities for investigating these questions in more flocks with and without cannibalism and under different environmental and management conditions. The detection system has to be further tested under the different farm conditions and to be advanced to a fully automatic surveillance system. In addition, more knowledge is needed regarding how cannibalism develops in a turkey flock and at which stages it can be influenced by management measures.

## 5. Conclusions

In this study, a newly developed pecking detection system that was based on a CNN model was successfully validated and tested on two turkey farms. The system showed a very satisfactory detection performance of around 90% or higher for all detection parameters (sensibility, specificity, accuracy, precision, and F1-score). It can be used to monitor object pecking under commercial conditions in the long-term, while it should be further tested under various management and housing conditions and be fully automated. Pecking frequencies were generally highest in the morning hours and overall showed low negative correlations with age during the rearing period, although increased frequencies could also be observed at the end of rearing and later in week 10 of life. The question of whether the onset of cannibalism can be detected by changes in object pecking frequencies could not be conclusively answered, partly due to a low sample size and partly due to lacking knowledge regarding the development of cannibalism in turkey flocks. The first indications that object pecking frequencies might reflect different levels of enrichment in the barn should, in addition, be investigated further.

## Figures and Tables

**Table 1:** Equation and definition of evaluation parameters.

Term	Equation/Definition
Sensitivity/Recall	$\frac{TP}{TP+FN}\times 100$
Specificity	$\frac{TN}{TN+FP}\times 100$
Accuracy	$\frac{TP+TN}{TP+TN+FP+FN}\times 100$
Precision	$\frac{TP}{TP+FP}\times 100$
F1-score	$2\times \left(\frac{Recall\times Precision}{Recall+Precision}\right)\times 100$
True Positive (TP)	observed pecking = recorded pecking
True Negative (TN)	observed non-pecking = recorded non-pecking
False Positive (FP)	observed non-pecking ≠ recorded pecking
False Negative (FN)	observed pecking ≠ recorded non-pecking

**Table 2:** Detection success of the convolutional neural network (CNN) model for female turkeys with intact beaks, *n* = 300 s (farm 1, weeks of life 2–14) and male turkeys with trimmed beaks, *n* = 120 s (farm 2, week of life 4 and 5).

Week	TP	TN	FP	FN	Sensitivity/Recall (%)	Specificity (%)	Accuracy (%)	Precision (%)	F1-Score (%)
Farm 1									
2	45	240	11	4	91.8	95.6	95.0	80.4	85.7
3	51	242	5	2	96.2	98.0	97.7	91.0	93.6
4	166	117	13	4	97.6	90.0	94.3	92.7	95.1
5	141	152	1	0	100.0	99.3	99.7	99.3	99.7
6	89	184	9	18	83.2	95.3	91.0	90.8	86.8
7	13	283	2	2	86.7	99.3	98.7	86.7	86.7
8	4	288	5	3	57.1	98.3	97.3	44.4	50.0
9	25	269	0	6	80.6	100.0	98.0	100.0	89.3
10	3	295	1	1	75.0	99.7	99.3	75.0	75.0
11	8	284	6	2	80.0	97.9	97.3	57.1	66.7
12	24	259	15	2	92.3	94.5	94.3	61.5	73.9
13	20	277	0	3	87.0	100.0	99.0	100.0	93.0
14	34	255	4	7	82.9	98.5	96.3	89.5	86.1
Total	623	3145	72	54	92.0	97.8	96.8	89.6	90.8
Farm 2									
4	23	86	1	10	74.2	98.9	90.8	95.8	83.6
5	42	69	5	4	91.3	93.2	92.5	89.4	90.3
