# Addendum: Gonzalez, J.J., et al. Automatically Detected Pecking Activity in Group-Housed Turkeys. *Animals* 2020, *10*, 2034

**DOI:** 10.3390/ani11030791

**Published:** 2021-03-12

**Authors:** Jennifer J. Gonzalez, Abozar Nasirahmadi, Ute Knierim

**Affiliations:** 1 Farm Animal Behaviour and Husbandry Section, University of Kassel, 37213 Witzenhausen, Germany; uknierim@uni-kassel.de; 2 Agricultural and Biosystems Engineering Section, University of Kassel, 37213 Witzenhausen, Germany; abozar.nasirahmadi@uni-kassel.de

The authors wish to add the following to this paper [1]:
